# The Roles of IL-1 Family Cytokines in the Pathogenesis of Systemic Sclerosis

**DOI:** 10.3389/fimmu.2019.02025

**Published:** 2019-09-13

**Authors:** Dan Xu, Rong Mu, Xiaofan Wei

**Affiliations:** ^1^Department of Rheumatology and Immunology, Peking University People's Hospital, Beijing, China; ^2^Key Laboratory of Carcinogenesis and Translational Research (Ministry of Education) and State Key Laboratory of Natural and Biomimetic Drugs, Department of Human Anatomy, Histology, and Embryology, Peking University Health Science Center, Beijing, China

**Keywords:** IL-1 family cytokines, systemic sclerosis, scleroderma, fibrosis, pathogenesis

## Abstract

The IL-1 family consists of 11 cytokines, 7 ligands with agonist activity (IL-1α, IL-1β, IL-18, IL-33, IL-36α, IL-36β, IL-36γ) and four members with antagonistic activities [IL-1 receptor antagonist (IL-1Ra), IL-36Ra, IL-37, IL-38]. Recent articles have described that most members of IL-1 family cytokines are involved in the process of innate and adaptive immunity as well as fibrosis in systemic sclerosis (SSc). IL-1 family gene polymorphisms, abnormal expression of IL-1 and its potential role in the fibrosis process have been explored in SSc. IL-33 and IL-18 have also been discussed in the recent years. IL-33 may contribute to the fibrosis of SSc, while IL-18 remains to be researched to confirm its role in fibrosis process. There is a lack of study on the pathophysiological roles of IL-36, IL-37, and IL-38 in SSc, which might provide us new study area. Here, we aim to give a brief overview of IL-1 family cytokines and discuss their pivotal roles in the pathogenesis of SSc.

## Introduction

Systemic sclerosis (SSc) is an idiopathic autoimmune disease characterized by immune dysfunction, vasculopathy, and progressive fibrosis in skin and internal organs. Clinically, skin thickening and fibrosis are the most typical features of SSc. In patients with SSc, major causes of premature death are the pathological changes in lung, gastrointestinal tract, kidney, heart (1). However, the etiology and pathogenesis of immune abnormalities and fibrosis in SSc are poorly understood, which leads to a lack of effective treatments for SSc. The current treatment is mainly non-specific symptomatic treatment, which can only temporarily improve the condition but cannot fundamentally control the progress of fibrosis (2).

The interleukin (IL)-1 family is a group of 11 proinflammatory and anti-inflammatory cytokines. Recent findings show that expression of most IL-1 family cytokines, such as IL-1α, IL-1β, IL-18, and IL-33, was abnormal in many autoimmune diseases including SSc. Similarly, gene polymorphisms of IL-1α, IL-1β, IL-18, and IL-33 were reported to be correlated with SSc susceptibility. Therefore, in this review, we provide a brief introduction of IL-1 family cytokines biological functions, the association of IL-1 family genes and SSc and the roles of IL-1 family cytokines in the expression and pathogenesis of SSc. The IL-1 family cytokines and their roles in SSc are summarized in Table 1.

**Table 1:** The role of IL-1 family members in the pathogenesis of SSc.

**Common name**	**IL-1 family name**	**Receptor**	**Co-receptor**	**Potential roles in SSc or fibrosis**
IL-1α	IL-1F1	IL-1R1	IL-1 RAcP (also termed IL-1R3)	Up-regulated in the lesional skin and serum. Induce the production of IL-6 and PDGF. Promote viability of SSc fibroblasts.
IL-1β	IL-1F2	IL-1R1 and IL-1R2	IL-1 RAcP	Elevated in the serum, BAL, and lesional skin. Induce IL-6 and TGF-β1, promote Th17 cell differentiation
IL-1Ra	IL-1F3	IL-1R1	NA	Up-regulated in SSc-affected fibroblasts. Induce fibroblasts differentiate into myofibroblast
IL-18	IL-1F4	IL-18Rα (also termed IL-18R1 or IL-1R5)	IL-18Rβ (also termed IL-1R7)	Elevated in serum and BAL. Pro-and anti-fibrotic effects were reported in fibrosis
IL-33	IL-1F11	ST2 (also termed IL-1R4)	IL-1 RAcP	Down-regulated in early SSc, upregulated in late SSc. Elevated in the serum. Induce M2 macrophages and ILC2 to produce IL-13 and TGF-β
IL-36α, IL-36β, IL-36γ	IL-1F6	IL-36R (also termed IL-1R6)	IL-1 RAcP	Drive fibrosis and activate the NLRP3 inflammasome
IL-36Ra	IL-1F5	IL-36R	NA	Unknown
IL-37	IL-1F7	IL-18Rα	SIGIRR (also termed IL-1R8)	Down-regulate pro-inflammatory cytokines
IL-38	IL-1F10	IL-36R	TIGIRR-2 (also termed IL-1R9)	Unknown

## The Biological Characteristics of IL-1 Family Cytokines

The IL-1 family consists of 7 members with agonistic functions (IL-1α, IL-1β, IL-18, IL-33, IL-36α, IL-36β, and IL-36γ) and 4 members with antagonistic activities, including IL-1Ra, IL-36Ra, IL-37, and IL-38 (3). IL-1 family cytokines are divided into 3 subfamilies based on the length of precursor protein and the N-terminal pro-pieces for each precursor. The IL-1 subfamily consists of IL-1α, IL- 1β, IL-33, and possess the longest pro-pieces, composed of ~270 amino acids. The IL-18 subfamily is comprised of IL-18 and IL-37 and also possess long pro-pieces composed of ~190 amino acids. IL-36 subfamily comprising IL-36α, IL-36β, IL-36γ, and IL-38 possess the shortest pro-pieces of ~150 amino acids (4).

Most IL-1 family members are commonly expressed as full-length precursors that require proteolytic processing for biologically mature forms. The full-length IL-1α is cleaved by the cysteine protease calpain, whereas IL-1β and IL-18 precursors require proteolytic cleavage by the inflammasome (5). IL-33 and IL-36 require neutrophil proteinases such as elastase and proteinase-3 for their processing (6, 7). IL-37 is cleaved by capsase-1 before maturation (8). IL-38 is bioactive as a full-length molecule.

IL-1 family cytokines activate signal transduction by the IL-1 receptor (IL-1R) family, which consists of 10 members: IL-1R1, IL-1R2, IL-1R accessory protein (IL-1RAcP), IL-18Rα, IL-18Rβ, ST2 (or IL-33R), IL-36R, single Ig IL-1R-related molecule (SIGIRR), three Ig domain-containing IL-1R related-2 (TIGGIR-2), and TIGGIR-1 (9). The receptor and co-receptor of IL-1 family are summarized in Table 1. With the exception of SIGIRR, which contains only one extracellular immunoglobulin (Ig) region, the other IL-1R members have three extracellular Ig regions. The intracellular domains of the IL-1R members are toll-like/IL-1R (TIR) domains. IL-1R2 is unique in IL-1R family because of lacking a TIR domain.

**Figure 1:**
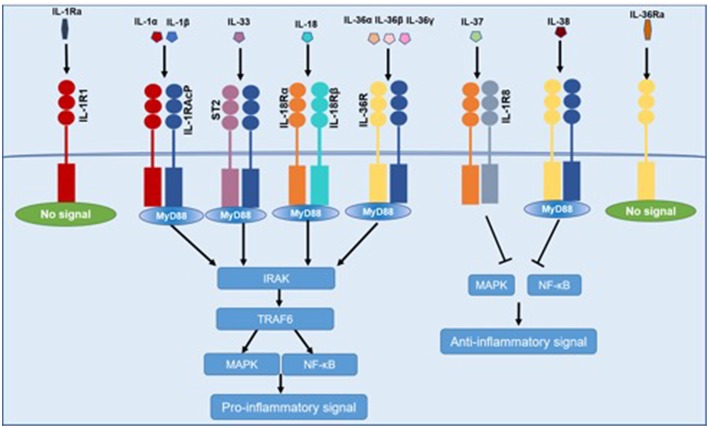
Common signaling pathway for IL-1 family cytokines. IL-1α, IL-1β, IL-18, IL-33, and IL-36 bind to IL-1R family members, recruiting MyD88, IRAK4, TRAF6, which resulted in the activation of NF-κB and MAPK and then promoting the transcription of several inflammatory genes. IL-37 and IL-38 exert anti-inflammatory effects by inhibiting NF-κB and MAPK signaling. IL-1Ra and IL-36 Ra cannot recruit the signaling chain.

Pro-inflammatory cytokines of IL-1 family (IL-1α, IL-1β, IL-18, IL-33, IL-36) bind to similar conserved receptors consisting of extracellular Ig domains and intracellular TIR domains and induce cell activation through recruiting cytoplasmic myeloid differentiation primary response protein 88 (MyD88), IL-1R associated kinase 4 (IRAK4), tumor necrosis factor receptor-associated factor 6 (TRAF6), which ends up in the activation of nuclear factor-κB (NF-κB), and mitogen-activated protein kinase (MAPK) (10). IL-37 binds to the IL-18Rα and subsequently recruits SIGIRR, which does not trigger the recruitment of MyD88 (11). IL-38 mainly binds to the IL-36R. Both IL-37 and IL-38 exert anti-inflammatory effects by inhibiting NF-κB and MAPK signaling (12). IL-1Ra and IL-36 Ra, in competition with IL-1α, IL-1β, and IL-36, respectively, bind to IL-1R1 and IL-36R, and cannot recruit the co-receptor, which eventually results in the inhibition of IL-1 and IL-36 signaling (Figure 1).

## The Association of IL-1 Family Genes and SSc

Different single nucleotide polymorphisms (SNPs) may result in the production of structurally different proteins with specific transcription rate and biological function. Investigating the correlation between SNPs of a specific gene and SSc seems to be inducible to understand the disorder's pathogenesis and find biomarkers for predicting the risk of SSc. In recent years, genome-wide association studies have revealed associations between genes encoding IL-1 family cytokines and SSc, which further supported the participation of IL-1 family cytokines in pathogenesis of the disease.

IL-1 family gene complex is located on chromosome 2q13–21. It consists of IL-1A, IL-1B, and IL-1RN. The human IL-1α (IL-1A) gene contains common SNPs including rs1800587 and rs17561, which have been reported to be linked to several autoimmune diseases in some populations. IL-1A rs1800587 was reported to be associated with SSc susceptibility in the Slovak Caucasian, Japanese, and Chinese populations (13–15). However, this association was not supported by other relevant studies (16). And in the meta-analysis, IL-1A rs17561 or rs1800587 polymorphism seems not to be statistically linked to the risk of SSc (17). Recently, a study demonstrated a significant association between the IL-1β (IL-1B) (+3962; rs1143634) SNP and the development of a severe ventilatory restriction in SSc patients. This SNP acted as an independent risk factor for restrictive lung disease along with the diffuse cutaneous subset of SSc and the presence of the anti-topoisomerase I antibody in a Cox regression model (18). However, in another study focused on Italian SSc patients, the frequency of the IL-1B rs1143634 CT genotype was significantly lower in patients with SSc compared to the control group, which indicated that this allele might be protective (16). Mattuzzi et al. described that IL-1BC-31C (rs1143627) and IL-1BC-511-T (rs16944) were significantly more frequent in SSc patients compared with controls (19). Also, Beretta determined the ability of epistatic interactions of cytokine SNPs to predict susceptibility to disease subsets in SSc. They performed the MDR analysis and showed a significant epistatic interaction among IL-1 receptor Cpst1970T, IL-6 Ant565G, and IL-10 C-819T SNPs increased the dcSSc susceptibility (20).

The association between IL-18 SNPs and SSc susceptibility was also analyzed. Results showed that IL-18 rs187238 and IL-18 rs1946518 polymorphism were not correlated with SSc susceptibility. However, ESR and dyspnea were associated with IL-18 rs187238 and IL-18 rs1946518 polymorphism, respectively (13).

Several studies focused on the association of genetic polymorphism of IL-33 in SSc patients. A multicentric preliminary study in 300 Turkish patients with SSc and 280 healthy controls showed that rs7044343 polymorphism of IL-33 gene was related to increased susceptibility to SSc (21). However, another study failed to find any association between IL-33 rs7044343 polymorphism and SSc susceptibility in Chinese population (13).

These results indicate that genetic variations of certain IL-1 family members are implicated in the pathogenesis of the disease and associate with SSc susceptibility. Table 2 summarizes the association between SNPs and SSc.

**Table 2:** Genetic polymorphisms in the IL-1 family cytokines that are associated with SSc.

**IL-1 family**	**SNP associated with SSc**	**Risk/protection**	**References**
IL-1A	rs1800587	Risk	(13–15)
	rs17561		(17)
IL-1B	rs1143634	Protection	(16)
	rs1143627	Risk	(19)
	rs16944	Risk	(19)
IL-18	rs1946518		(13)
	rs187238		(13)
IL-33	rs7044343	Risk	(21)
	rs1157505		(13)
	rs11792633		(13)
	rs1929992		(13)

## The Expression and Function of IL-1 Family Cytokines in SSc

### The Expression and Function of IL-1α in SSc

The expression of IL-1α and IL-1β mRNA were barely detectable in unstimulated dermal fibroblasts, however, their expression was strongly increased after adding IL-1α and TNF-α. Cultured dermal fibroblasts from patients with SSc expressed higher levels of intracellular IL-1α than fibroblasts from healthy subjects (22). Immunohistochemical analysis showed that the expression of intracellular IL-1α was constitutively up-regulated in the lesion skin fibroblasts of SSc patients. The production of pro-collagen and IL-6 were decreased when the expression of IL-1 α was inhibited via IL-1α siRNA in SSc-affected fibroblasts. Conversely, overexpression of IL-1α through stable transfection in normal fibroblasts induced the differentiation of the SSc fibroblast phenotype (23). These evidence suggested that IL-1α could have a potential role in regulating fibroblast–myofibroblast differentiation, which is believed to be a key event in SSc. In addition, the serum level of IL-1α in SSc is controversial. Lin et al. reported that SSc patients with high serum IL-1α concentrations were more likely to have digital ulcers (24). These data emphasize the need for further research to determine the role of IL-1α in SSc pathogenesis, particularly in obliterative vasculopathy.

Endogenous IL-1α can induce fibroblast proliferation and collagen production through promoting the production of IL-6 and platelet-derived growth factor (PDGF) in SSc. Then, inhibition of endogenous IL-1α resulted in the decreased expression levels of IL-6 and PDGF in SSc fibroblasts (25). IL-6 is a critical mediator of fibrosis in SSc via inducing pro-fibrotic gene expression *in vivo*, enhancement of TGFβ1 production and by regulating TGFβ receptor (26, 27). TGF-β1 is a major regulator of fibrosis through stimulating cells undergoing epithelial-mesenchymal transition (EMT), fibroblast proliferation, ECM synthesis, and inhibition of collagenase and matrix metalloproteinases (MMP) (28, 29).

As mentioned above, IL-1α also induced PDGF, a potent chemotactic factor for inflammatory cells and TGF-β1, which can directly induce the differentiation of fibroblasts into actively EMC-producing myofibroblasts (30). What's more, in SSc fibroblasts, IL-1α can bind nuclear protein necdin in SSc fibroblasts and antagonize the function of necdin, which has an inhibitory effect on procollagen type I production (31). In addition, IL-1α and IL-1β were found to promote the viability of cultured fibroblasts and myofibroblasts from patients with SSc and this directly induced expression of N-cadherin and α-SMA, which is commonly used as a specific marker of myofibroblasts formation (32). These findings showed that IL-1 might contribute to fibroblast–myofibroblast differentiation and the myofibroblasts longevity, which are believed to be key events in SSc consequent skin fibrosis in patients with SSc.

In animal models of allergy, IL-1α and IL-1β were required for Th2 cell activation during airway hypersensitivity response (33). IL-1α and IL-1β were also proved to sustain the Th2 immune responses in parasites infestation (34). However, very few studies focused on the effects of IL-1α either IL-1β in Th2 cells in SSc. Considering the pathogenic role of Th2 cells in SSc, we would have expected to find many papers studying the pathogenic roles of IL-1α and IL-1β in SSc patients.

### The Expression and Function of IL-1β in SSc

In patients with SSc, studies have observed a distinct elevation of IL-1β in the serum and bronchoalveolar lavage fluid (BAL) (35). In the lesion skin tissue of SSc patients, the expression levels of IL-1β and IL-18 were significantly up-regulated. Furthermore, there was a positive association between dermal fibrosis severity evaluated by modified Rodnan skin score (MRSS) and IL-1β and IL-18 expression, respectively (36). IL-1β has been abnormally expressed in a variety of fibrotic diseases. Studies have showed that pulmonary fibrosis induced by bleomycin, renal interstitial fibrosis resulting from unilateral ureteric obstruction, liver fibrosis in hypercholesterolemic and cardiovascular fibrosis after myocardial infarction are all attenuated in IL-1β-deficient mice (37–40).

Like IL-1 α, IL-1β also induces myofibroblast activation and fibrosis through IL-6 and TGF-β1. In addition, it has been observed that IL-1β and TGF-β2 can drive endothelial to mesenchymal transition (EMT), which is an important pathologic process in fibrosis (41). IL-1β has also been found to participate in the differentiation of Th17 cells that may play a crucial role in the development of tissue fibrosis (42).

What's more, the upstream regulation of IL-1β also has effects on the pathogenesis of SSc. The inflammasome has been found to be important in the pathogenesis of SSc by activating some IL-1 family cytokines such as IL-1β and IL-18. Inflammasomes are poly-protein complexes. Many subtypes of inflammasomes have been identified, and the nucleotide-binding domain, leucine rich repeat containing family and pyrin domain-containing 3 (NLRP3) inflammasomes are the most extensively studied in SSc. The critical function of the NLRP3 inflammasome is to activate caspase-1, which can cleave the precursors of IL-1β and IL-18 into biologically active forms. Inhibition of caspase-1 could reduce the secretion of IL-1β and IL-18 in SSc skin and lung fibroblasts. In addition, the expression of α-SMA protein was decreased in SSc dermal myofibroblasts when treated with a caspase-1 inhibitor. Furthermore, NLRP3^−/−^ mice were resistant to bleomycin-induced skin fibrosis (43). One study observed the significantly increased expression of NLRP3, caspase-1, IL-1β, IL-18, and a positive correlation between the severity of dermal fibrosis and NLRP3 inflammasome in SSc lesion skin (36). Mechanistically, Artlett et al. reported that inflammasome promoted the expression of miR-155, which is critical in driving fibrosis in SSc (44). Overall, NLRP3 inflammasome and its subsequent effectors have been proven to be critical in the development of SSc, and deemed as promising candidates for targeting treatment in the clinics.

### The Expression and Function of IL-Ra in SSc

IL-1Ra comprises 4 different isoforms. One isoform (sIL-1Ra) is secreted and the other three (icIL-1Ra1, icIL-1Ra2, and icIL-1Ra3) are intracellular. Both sIL-1Ra and icIL-1Ra1 mRNAs were constitutively expressed by human dermal fibroblasts, whereas intracellular IL-1Ra was markedly up-regulated in SSc-affected fibroblasts compared to normal skin fibroblasts after stimulating with IL-1 β or TNF-α (22). When Intracellular IL-1Ra is overexpressed in cultured normal human skin fibroblasts via transfection with a viral vector, it induces a myofibroblasts phenotype characterized by increased expression of α-SMA and plasminogen activator inhibitor-1 (PAI-1), which plays a crucial role in fibrogenesis and is expressed markedly in myofibroblasts, along with decreased expression of collagenase and MMP-1 (an enzyme involved in the breakdown of ECM in the skin) (45). Collectively, these studies suggested that intracellular IL-1Ra might be relevant to the pathogenesis of fibrosis in SSc.

### The Expression and Function of IL-33 in SSc

Studies showed that IL-33 played an important role in the pathogenesis of multiple autoimmune diseases, such as systemic lupus erythematosus (SLE), rheumatoid arthritis (RA) and inflammatory bowel disease (IBD) (46–48). Recently, an increasing number of studies have shown the potential role of IL-33 in SSc. In the skin biopsies from early SSc patients, the expression of IL-33 protein was down-regulated. By contrast, in patients with late stage SSc, IL-33 protein was constitutively found in most endothelial cells (49). Several studies demonstrated that serum level of IL-33 was elevated in patients with SSc compared with healthy controls. High serum level of IL-33 was positively correlated with peripheral vascular involvement, such as digital ulcers and the severity of skin sclerosis and pulmonary fibrosis (50–52).

**Figure 2:**
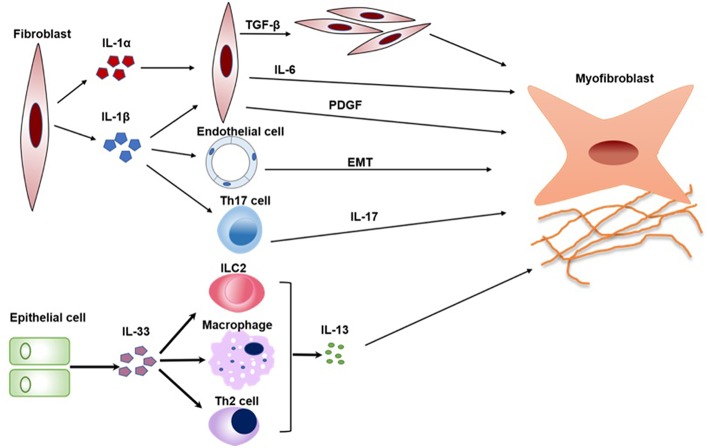
The pathogenic roles of IL-1 family in SSc. Key mechanisms by which IL1α, IL-1β, and IL-33 promotes a variety of cells activation and transition are summarized in a schematic form. IL-1α and IL-1β can induce fibroblast proliferation and fibrosis through promoting the production of IL-6 and PDGF. In addition, IL-1β can also promote EMT and the differentiation of Th17 cells, which both play crucial roles in SSc. IL-33 induces the expansion of Th2 cells, ILC2, and M2 macrophages to increase the production of IL-13. IL-13 is a profibrotic cytokine that is sufficient for the induction of fibrosis in SSc. PDGF, platelet-derived growth factor; EMT, epithelial-mesenchymal transition; ILC2, type 2 innate lymphoid cell.

When IL-1RAcP^−/−^, ST2^−/−^, and wild-type (WT) mice were treated by recombinant IL-33, IL-1RAcP^−/−^, and ST2^−/−^ mice did not develop cutaneous fibrosis compared to WT mice, which means that IL- 33 induces cutaneous fibrosis by type 2 immunity is ST2 and IL-1RAcP-dependent (53). IL-33 can participate in the polarization of M2 macrophages to produce IL-13 and TGF-β1, which are both profibrotic cytokine in pathological fibrosis (54). In addition, IL-33 also induced the expansion of type 2 innate lymphoid cells (ILC2s) to increase the production of IL-13 (55) (Figure 2).

In brief, the critical role of IL-33 in SSc pathogenesis has been elucidating. However, more studies on the precise function of IL-33 in the process of immune dysfunction, vasculopathy, and fibrosis are required in SSc.

### The Expression and Function of IL-18 in SSc

Serum IL-18 levels in SSc patients were significantly higher than that in control subjects and positively correlated with the presence of anti-nuclear antibody (ANA) and clinical grades in patients with SSc, respectively (56). IL-18 levels in serum and BAL in patients with IPF were also increased compared with control subjects (57). These results indicated that IL-18 may be involved in the process of fibrosis. However, the exact mechanism of the IL-18 in fibrosis is controversial because both pro-and anti-fibrotic effects were reported in the literature.

Kitasato et al. reported that IL-18 mediates hepatic fibrosis by activating CD4+ T cells, and that this effect is blocked by anti-IL-18 treatment. Moreover, in renal fibrosis, stimulating proximal tubular cells with IL-18 could induce α-SMA, collagen I, and fibronectin production in a dosage and time dependent fashion (58).

On the contrary, some studies have observed that IL-18 has anti-fibrotic effects. Nakatani-Okuda et al. reported that mice deficient in IL-18 developed more severe fibrosis than WT mice (59). Furthermore, Kim et al. demonstrated that IL-18 down-regulated the production of collagen in human dermal fibroblasts through the E26 transformation-specific-1 and the ERK pathway, indicating that IL-18 may have anti-fibrotic effects in patients with SSc (60). Whether IL-18 has pro-fibrotic or anti-fibrotic effects need further validation.

### The Pathogenesis of IL-36, IL-37, and IL-38 in SSc

At present, increasing number of studies suggested important roles of IL-36, IL-37, and IL-38 in a variety of autoimmune diseases. However, few studies have evaluated their expression and pathophysiological roles in SSc patients. Thus, information obtained from studies of other autoimmune and fibrotic diseases may be beneficial to understand their potential effects on SSc.

IL-36 comprises 3 isoforms, IL-136α, IL-36β, and IL-36γ. At present, very limited evidence in the researches regarding IL-36 in SSc or fibrosis has been reported. IL-36α was observed to induce tubulointerstitial fibrosis in the mice model with unilateral ureteral obstruction. In IL-36 receptor knock-out mice, fibrosis was attenuated (61). In this study, recombinant IL-36α can activate the NLRP3 inflammasome. IL-36α was also elevated in the fibrotic tissue of patients with chronic pancreatitis, which further implicated IL-36 as a profibrotic cytokine (62). Several studies had shown that IL-36 was related to autoimmune diseases such as RA, SLE, and psoriasis (63, 64).

Low doses of IL-37 inhibited joint inflammation and significantly decreased synovial IL-1β, TNF-α, IL-6, CCL3, CXCL1 in an arthritis mice model (65). IL-37 also played an effective immunosuppressive role in experimental psoriasis by down-regulating pro-inflammatory cytokines such as IL-6, TNF-α, and IL-1β (66). No data about IL-37 and fibrosis has been reported so far.

IL-38 seems to play a role in the development of fibrosis. The expression of IL-38 is significant in the lungs of patients with acute idiopathic pulmonary fibrosis (67). However, further investigation is needed to explore their potential roles and their receptors in SSc.

In conclusion, the functional implications of IL-36 and IL-38 are not yet known in SSc, but similar studies in tubulointerstitial fibrosis and IPF have indicated that IL-36 and IL-38 may induce fibrosis. A similar understanding in SSc would represent a significant advance. IL-37 down-regulates pro-inflammatory and pro-fibrotic cytokines such as IL-6 and IL-1β. Therefore, whether IL-37 could play immunosuppressive and anti-fibrotic roles in SSc requires further study.

## Clinical Application Via Inhibiting IL-1 Family Cytokines in SSc

In recent years, clinical application targeting IL-1 family cytokines has been used in multiple autoimmune diseases such as RA and gout (68, 69). However, few studies have explored the clinical benefits in patients with SSc.

In a clinical trial, rilonacept, an IL-1 receptor fusion protein, did not show treatment-related efficacy in patients with SSc compared to placebo, and also failed to reduce the expression of IL-6, C-reactive protein (CRP), or CCL18 expression (70). Although anti-IL-1 therapy seems to be ineffective according to limited studies, the development of new biologics with specific IL-1 antagonists and the blocking of IL-18 or IL-33 may show potential clinical usefulness in the future.

## Conclusion

The IL-1 family of cytokines have been shown to play a vital role in the pathogenesis of SSc, and the IL-1 family gene polymorphisms have been demonstrated to be closely related to SSc. Recent studies have investigated the abnormal expression of IL-1 and its potential role in the fibrosis process. However, many aspects of IL-1 family members in SSc remain to be elucidated. There is large room for the mechanism study of IL-1 family cytokines, especially for IL-37 and IL-38. Furthermore, researches exploring the potential benefits of simultaneously inhibiting multiple members of the IL-1 family cytokines *in vivo* are promising.

## Author Contributions

DX prepared the draft manuscript. RM and XW revised and finalized the article. All authors have read and approved it for publication.

### Conflict of Interest Statement

The authors declare that the research was conducted in the absence of any commercial or financial relationships that could be construed as a potential conflict of interest.
